# Intra-hospital organ and tissue donation coordination project: cost-effectiveness and social benefits

**DOI:** 10.1590/S0034-8910.2015049005770

**Published:** 2015-10-05

**Authors:** Vanessa Silva e Silva, Luciana Carvalho Moura, Renata Fabiana Leite, Priscilla Caroliny de Oliveira, Janine Schirmer, Bartira De’ Aguiar Roza

**Affiliations:** I Programa de Pós-Graduação em Enfermagem. Escola Paulista de Enfermagem. Universidade Federal de São Paulo. São Paulo, SP, Brasil; II Instituto Israelita de Responsabilidade Social. Hospital Israelita Albert Einstein. São Paulo, SP, Brasil; III Departamento de Enfermagem na Saúde da Mulher. Escola Paulista de Enfermagem. Universidade Federal de São Paulo. São Paulo, SP, Brasil; IV Departamento de Enfermagem Clínica e Cirúrgica. Escola Paulista de Enfermagem. Universidade Federal de São Paulo. São Paulo, SP, Brasil

**Keywords:** Tissue and Organ Harvesting, Patient Care Team, Nursing, manpower, Costs and Cost Analysis, Cost-Benefit Analysis

## Abstract

**OBJECTIVE:**

To evaluate the viability of a professional specialist in intra-hospital committees of organ and tissue donation for transplantation.

**METHODS:**

Epidemiological, retrospective and cross-sectional study (2003-2011 and 2008-2012), which was performed using organ donation for transplants data in the state of Sao Paulo, Southeastern Brazil. Nine hospitals were evaluated (hospitals 1 to 9). Logistic regression was used to evaluate the differences in the number of brain death referrals and actual donors (dependent variables) after the professional specialist started work (independent variable) at the intra-hospital committee of organ and tissue donation for transplantation. To evaluate the hospital invoicing, the hourly wage of the doctor and registered nurse, according to the legislation of the Consolidation of Labor Laws, were calculated, as were the investment return and the time elapsed to do so.

**RESULTS:**

Following the nursing specialist commencement on the committee, brain death referrals and the number of actual donors increased at hospital 2 (4.17 and 1.52, respectively). At hospital 7, the number of actual donors also increased from 0.005 to 1.54. In addition, after the nurse started working, hospital revenues increased by 190.0% (ranging 40.0% to 1.955%). The monthly cost for the nurse working 20 hours was US$397.97 while the doctor would cost US$3,526.67. The return on investment was 275% over the short term (0.36 years).

**CONCLUSIONS:**

This paper showed that including a professional specialist in intra-hospital committees for organ and tissue donation for transplantation proved to be cost-effective. Further economic research in the area could contribute to the efficient public policy implementation of this organ and tissue harvesting model.

## INTRODUCTION

Brazil has the largest public health system in the world and, in the case of organ transplants; it is unique concerning its funding model. This situation is a result of public movements in the 1970/80s that precipitated the creation of the Unified Health System (SUS), in 1990, which is regulated by the *Lei Orgânica da Saúde* (Organic Health Law) 8.080.^a^ The SUS has been defined as “set of activities and services at all direct or indirect administration levels (local, state and federal) that are maintained by the Brazilian Public Authority”.^14^

After the SUS had been in existence for a few years, ordinances and resolutions were designed to establish values for financing health procedures. However, 1999 saw actions relating to donating and transplanting organs made easier with the publication of Ordinance 531/GM,^b^ which created the *Fundo de Ações Estratégicas e Compensação *(FAEC – Strategic Actions and Compensation Fund); this was supplemented with Ordinance 510 in 2010,^c^ which changed the values referring to transplant-related activities. These values, which are stored in a unified table of procedures, medicines, orthotics and prosthetics, are annually reviewed and updated by means of new Ordinances.^d^

Along with this historical evolution in the law, the public financial budget earmarked for activities related to organ donation and transplantation increased.

In spite of these investments, some professionals work in the area, e.g., in *comissões intra-hospitalares de doação de órgãos e tecidos para transplantes* (CIHDOTT – Intra-hospital Committees of Organ and Tissue Donation for Transplantation), who do not exclusively perform this activity. There are unpaid professionals for this function who do this alongside other functions at the hospital.

In most cases, the Coordinator of the CIHDOTT is the nurse, whose expertise in this area is regulated by the COFEN Resolution 292/2004.^e^ This resolution describes the management of the whole process of organ donation as the nurse’s responsibility, from identifying the potential donor to delivering the body to the family.

One charity hospital in Sao Paulo has developed an intra-hospital organ and tissue donation for transplantation coordination project for public hospitals, in partnership with the National Transplant System and the Secretariat of Health for the State of Sao Paulo in 2008, and performed by the Institutional Development Support Program of the SUS.^12^

This project encourages adherence of the law for implementing CIHDOTT in Brazil,^f^ through the aggregation of specialized human resources. The objective is to increase the number of organ and tissue donors and, thus, reduce spending on patients waiting for transplants. To understand this strategy’s results, the project was evaluated after five years.

The actual investment of the project takes the form of providing a health care professional who is an expert in the field, at no cost to the selected hospitals that host the project. In addition, these professionals add more value to hospital revenue, due to billing procedures related to organ donation, which are paid by the SUS table.

The objective of this study was to evaluate the feasibility of a professional specialist in Intra-hospital Committees of Organ and Tissue Donation for Transplantation.

## METHODS

This is an epidemiological study, both retrospective and transversal, performed with organ donation data from the Secretariat of Health for the State of Sao Paulo and the intra-hospital organ donation coordination project of a beneficent hospital, from 2003 to 2012.

The study population was made up based on the potential donor referrals and actual donor referrals from the state of Sao Paulo, with a sample before and after the nursing specialist’s arrival at nine hospitals, the selection criterion for which was to have at least six months experience as an intra-hospital coordinator hired by the intra-hospital coordination project for organ donation at the charity hospital.

The data, collected during one single occasion, were separated into two groups: one before the beginning of the project (September 2003 to September 2011) and one after (May 2008 to December 2012), with an equal number of months being evaluated before and after the project was implemented at each hospital.

The collection instrument was developed using a spreadsheet (Microsoft Excel^®^) with variables related to the number of brain death referrals and actual donors at the nine hospitals in which the project was hosted, numbering the hospitals from 1 to 9 to ensure anonymity. The numbers of referrals and actual donors were transcribed from the organ donation project database to the spreadsheet and were validated with official information from the database of the Secretariat of health of the state of Sao Paulo.

The instrument was built based on the researcher’s experience and was presented at the Study group in Organ Donation and Transplantation of the Universidade Federal de São Paulo (GEDOTT – UNIFESP). All suggestions were considered while developing the final instrument.

Logistic regression, which estimated the effect of time (month to month) on the number of brain death referrals and the number of actual donors, was used. This analysis made it possible to analyze the influence of professional activities on the hospitals’ results. The differentiated response pattern took place after the specialist nurse arrived at the hospitals in question, i.e., this was the ground zero of the regression.

The model was considered significant when p < 0.05. The coefficient of determination (R^2^) was used, as it is a measurement for fitting the generalized linear statistical model to the observed values. The R^2^ varies between 0 and 1 and indicates the fit quality of the model: the closer to 1, the better the fit.^g^ The results were presented in linear graphs, which presented a tendency to increase (rising line) or decrease (declining line) the events (brain death referrals and actual donors).

For the financial analysis, information from the invoicing procedures for organ donation from the Management System of the SUS Table of Procedures, Medications and Orthoses, Prostheses and Special Materials (SIGTAP^®^) were used.^d^ These values are available in the public domain and were multiplied by the number of actual donors before and after the project, at all the hospitals.

The following procedures from the SUS table, invoiced by the CIHDOTT, were considered to calculate the values: brain death evaluation, surgical room coordination, ICU-related daily allowance, family interview and potential donor maintenance.

Ordinance 2.600/2009 describes, in article 15 of section II of chapter III, that:

[...] a CIHDOTT must [...] consist of at least three members of staff, one of which must be a doctor or a nurse who will be the Intra-Hospital Organ and Tissue Donation for Transplantation Project Coordinator.

[...] §2 In hospitals with a CIHDOTT, classified as II and III, according to art. 14 of this Regulation, the Coordinator from the Intra-Hospital Organ and Tissue Donation for Transplantation Committee must spend a minimum of twenty hours per week dedicated exclusively to this Committee.

§ 3 the CIHDOTT Coordinator, classified as III, must be a medical professional. § [...].^h^

Thus, the mean salaries of the doctor and the nurse, based on information from their respective unions, were investigated. The values were submitted to hourly wage calculation, determined by the *Consolidação das Leis do Trabalho *(CLT – Brazilian Consolidation of Labor Laws),^i^ to estimate the salary figures, from a 20-hour working week (80 hours/month), for these professionals, which is as follows:

Hourly wage = monthly salary/No. hours per day x 30

The number of working hours per day was calculated as follows:

Daily working hours = weekly working hours/six days

(the monthly salary refers to six paid days and one day off per week)

The amount paid per month for a 20-hour working week is calculated by multiplying the hourly wage by 80 hours.

There was subsequently a discussion regarding whether the invoiced values from the SUS table could cover the cost of a professional exclusively working in intra-hospital committees. The salaries were considered with linear distribution, without specific calculations for analysis of variations over months or years. This is because it is the initial analysis of the project, with the limitation of the study being acknowledged.

Using financial metrics, such as return on investment (ROI) in health program studies, is discussed in the literature and refers to basic analysis of return on investment in health. We did not intend for this to be an applied analysis; however it helps to guide project administrators in the field of health regarding its continuation.^j^ Further analysis will be required to verify the specific costs, since most studies use formulas that have been adapted to the reality of the study.^8^ ^-^ ^10^ ^,^ ^13^ ^,^ ^15^

The ROI can be defined by the fraction: the numerator of which is the ‘net gain’, return, profit or benefit earned as a result of the project, activity or system; while the denominator is the cost (investment) assigned to achieve the result.^3^

The real investment for this project was the human resource (converted into US dollars), which was estimated based on the mean values calculated for a 20-hour working week, according to CLT.

The salaries were paid to professionals from the budget earmarked for the Institutional Development Support Program of the SUS and did not appear on the payroll of the selected hospitals. These salaries can be referred to as forms of investments, depending on the context.^5^

The SUS is not looking to gain profit, but to provide complete care to the user. Depending on the cost of performing the procedures and on salaries paid, there may or may not be economic profit.^12^

The net profit considered here was the result of subtracting the invoicing from the procedures with organ donation and from the costs that are inherent in the donation process; the first represents the direct result from the work of the professional. The costs were based on the one study conducted in the area that considered every step of the process up to the delivery of the body to the family.^6^

The ROI calculation considers the net income divided by the cost of the project. The higher the ROI, the greater the return on investment, according to the formula:^3^ ^,^ ^5^


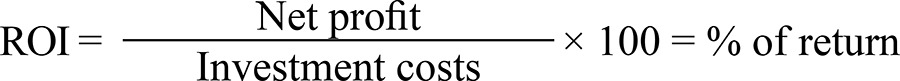


The other analysis performed was the Payback Period (PP) - *Prazo para Retorno do Investimento (PRI)*:^k^


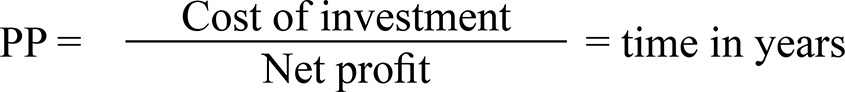


## RESULTS

The logistic regression models for brain death referrals presented a poor fit (p > 0.05), except for hospitals 1, 2 and 5, which were then descriptively analyzed. Whereas, for the number of actual donors, the model was well fitted for hospitals 2, 3 and 7, of the nine hospitals.

There was statistical significance in the increase of brain death referrals and actual donors from when the specialist nurse started at hospital 2 (increase of 4.17 brain death referrals and 1.52 actual donations compared to the previous month), as there was for actual donors in hospital 7 (1.54).

**Figure 1:**
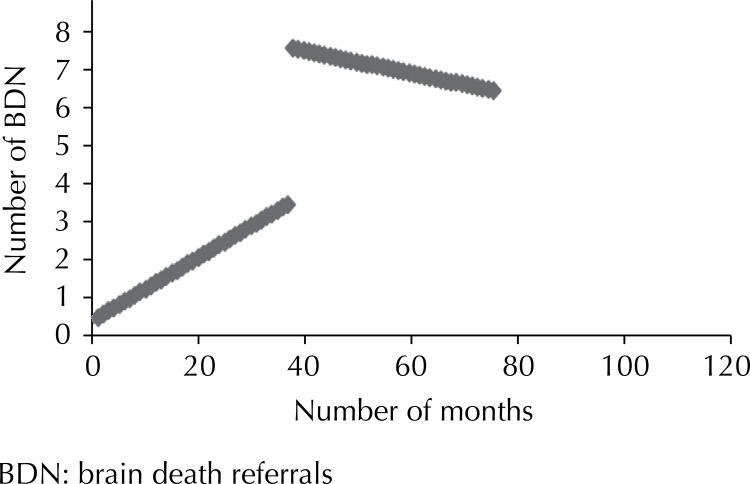
Number of brain death referrals before and after the project at Hospital 2. Sao Paulo, Southeastern Brazil, 2005 to 2011.

There was an increasing tendency with regard to brain death referrals only for hospital 9, with no statistical significance (Figures 1 [Fig f02] to 3). The other hospitals showed a downward tendency, with statistical significance in hospitals 5 and 9.

**Figure 2 f02:**
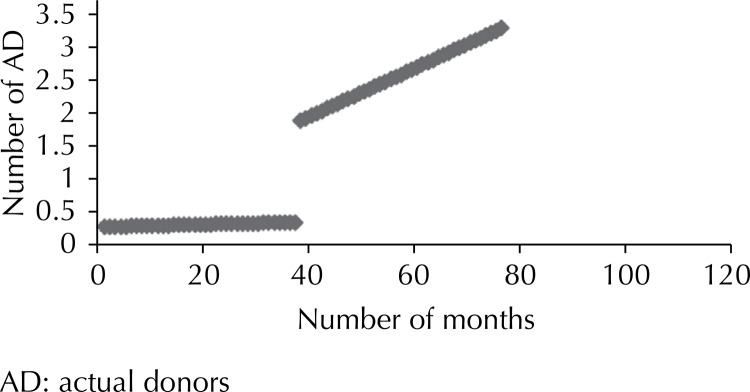
Number of actual donors before and after the project at Hospital 2. Sao Paulo, Southeastern Brazil, 2005 to 2011.

**Figure 3:**
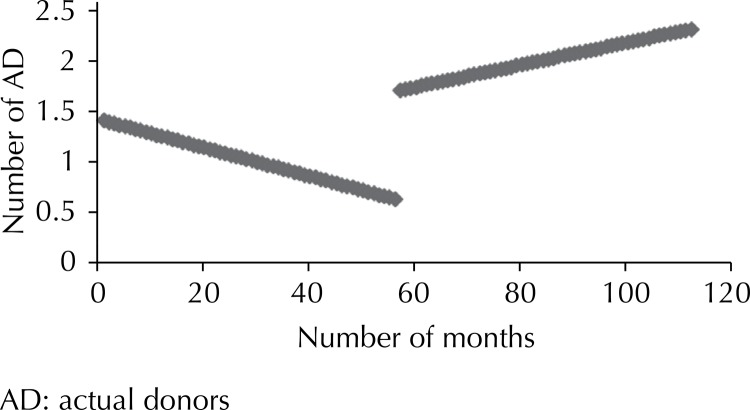
Number of actual donors before and after the project at Hospital 1. Sao Paulo, Southeastern Brazil, 2003 to 2012.

As regards the number of actual donors, an increasing trend was observed in four hospitals (1, 2, 5 and 9) and, in the others, a descending trend (3, 4, 6, 7 and 8), all with no statistical significance.

Hospital invoicing increased during the period, when the sum of month by month actual donors, multiplied by the corresponding value from the SUS table in Brazilian reals and in dollars, were considered:^l^ US$109,904.31 of invoicing before the project and US$315,708.91 after (difference of US$205,804.60 or 187.0%). Net income was US$237,644.62. Hospital invoicing increased at all the hospitals after the project was implemented, with a 40.0% to 1.955% variation.

The mean doctor and nurse salaries in the State of Sao Paulo were investigated, as described in the methods section of this article. The wage floor of a doctor working 20 hours per week, according to the *Federação Nacional dos Médicos *(FENAM – Brazilian National Federation of Doctors),^m^ was US$4,408.13 per month. The wage floor of a nurse, calculated by the mean wage floors from the unions in the State of Sao Paulo,^n^ was US$895.70 for 36 working hours per week.

The hourly wage, according to the CLT, was:

Doctor hourly wage = US$4,408.13/100 h = US$44.08/h

Nurse hourly wage = US$895.70/180 h = US$4.98/h

The Coordinator of one CIHDOTT type II can be a nurse, so the monthly cost would be US$398.40 for a 20-hour working week.

For CIHDOTT type III, there would be a monthly cost of US$3,526.40 to have a doctor coordinator working exclusively for 20 hours per week.

In the hospitals studied, the professionals were nurses. Therefore, the total value invested for salaries was US$91,134.63, considering the number of project months in each hospital (56 months in hospital 1; 39 in hospital 2; 8 in hospital 3; 17 in hospital 4; 20 in hospital 5; 20 in hospital 6; 26 in hospital 7; 28 in hospital 8; and 15 in hospital 9).

Based on the presented investment values and net income, the ROI and the PP were:


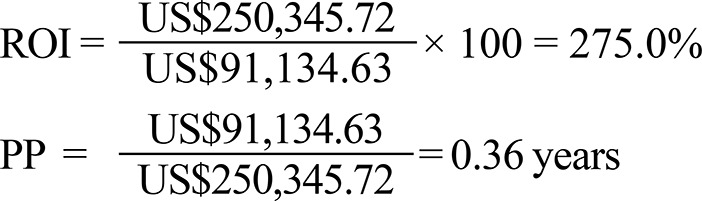


## DISCUSSION

The results for the number of actual donors in hospitals after the professional began work were positive. This confirms the findings of publications that discuss the proper operation of one CIHDOTT as being of paramount importance to increase the number of donors.^16^

Improving the processes involved with organ donation by means of there being a CIHDOTT ensures better quality and quantity of organs supplied to Brazil’s public transplantation system.^2^

Improving the number of brain death referrals and actual donors can ensure fairness, which is the guiding principle of the SUS, and help the patients return to the labor market, since many of these individuals rely on state benefits during their pre-transplant phase to live. Additionally, as the indirect costs of not performing transplants are high,^4^ ^,^ ^11^ this improvement would still decrease government spending on health, thereby allowing resources to be reallocated in hospitals and applied in sectors such as the intra-hospital committees of organ and tissue donation for transplantation.

Despite the pattern in the number of brain death referrals or actual donors having remained higher than in the period before the project’s implementation, there was a downward trend in the line – with their being exceptions in four hospitals.

A prolonged stay in the same hospital as was observed in hospitals 1 and 2, which can result in professional exhaustion. Studies relate high rates of Burnout Syndrome with professionals in the organ procurement area, which can range from emotional exhaustion and depersonalization to reduced personal job satisfaction.^1^

Psychological support and rotating professionals among the different hospitals are needed so that the reality of death is not the main aspect that is focused upon, but more the prevalence of life and the positive aspects that organ procurement provides.^7^

The presence of a coordinator with a workload and remuneration that are exclusive for organ donation activities is not a reality in many places in Brazil, such as in the State of Sao Paulo, as the project presented in this study shows. However, despite these initiatives, no regulations sustain this remuneration, by hospitals or by the Government, for intra-hospital coordinators.

Additionally, the diagnosis of brain death represents the death of the individual. Maintaining an organ donor is a costly activity, requiring specialist staff and hospital supplies. Therefore, the SUS created the ICU-related daily allowance for potential organ donors to ensure that the post-mortem phase of these is financially provided for.

The professional at one CIHDOTT follows the brain death cases from actively investigating coma patients, at level 3 on the Glasgow coma scale, until a potential donor becomes actual or not. This means that the family of the donor is dealt with sensitively, while also ensuring the family’s right to decide on organ and tissue donation (depending in the will of the deceased) and improve the turnover of hospital beds.

The turnover rate of beds in these cases refers to two outcomes: with the family acceptance for organ donation where the bed is released when the donor leaves the ICU to the operating room for the organ retrieval; and when the family refuses to donate the organs, the bed is subsequently released after the withdrawal of mechanical and drug support by the physician in charge to deliver the body to the family, who then proceed with the funeral ceremonies. Both cases present a social benefit in the form of a bed being made available, which ensures the possibility of another individual receiving therapy. In addition, the shorter the time taken to arrive at the outcome, the lower the costs are concerning human resources, medications, devices, therapeutic gases, among others.

Hospital invoicing is another point that deserves highlighting because the SUS allocates a specific budget to pay for procedures related to organ donation. Thus, the trained professional can assist in the hospital invoicing process and increase the amounts collected for the hospital.

The organ donation invoicing procedures in the hospitals studied contributed at least 40.0% more to hospital revenue than before the professional began work in the CIHDOTT. This benefit might be better explained in future analyses that use tools from Health Economy (*Economia da Saúde*), such as analyzing cost-effectiveness, seeing that “an intervention in health is said to be cost-effective if it produces a clinical benefit that is justifiable for its cost”.^o^

Organ donation must be self-sustaining to compensate for the social benefit to the individual, who only has this option as a possible therapy. Hospital invoicing procedures, which result from procedures performed with potential donors and actual organ donors (ICU daily allowance, family interview, operate room coordination, among others), could make covering their own expenses with the potential donor (labor from nursing professionals, medication use and therapeutic gas use, among others) and by hiring a professional to carry out the intra-hospital coordination their objective.

By charging for all the items listed in the method, the donor could generate revenues of US$635.52, according to SUS table. Thus, by only considering the salary costs of a doctor coordinator for CIHDOTT type III cases, an average of 3.7 donors would be required per month with the charging of all the previously listed items. One donor per month would be enough to cover salaries at 20 hours per week for the cost of a CIHDOTT type II nurse coordinator.

Hiring nurses to coordinate CIHDOTT type III could be an economically viable alternative to these hospitals, based on *Economia da Saúde*. The transplant system’s structure in the State of Sao Paulo relies on the support of the Organ Procurement Services, which have professional doctors as coordinators who validate the organ donors and provide assistance during the donation process. In addition, many activities carried out by CIHDOTT professionals are the nurses’ responsibility, who would guarantee the safety and quality of the process within the hospital.

Spending with the basic monthly salary of this coordinator, at 20 hours per week, represents 11.3% of the salary that a doctor would earn. To do this, there would need to be calculated studies and possible changes in the current legislation.

In hospitals with nurse coordinators, efficient use of funds applied in the salaries of nurses in the project was made, with an ROI of 275.0% and a short period for a return on the investment: less than one year (PP = 0.36 years). These economic indicators evaluate the financial impact and are also useful for public managers’ decision-making processes. However, these indicators need to be applied more fully in further studies to have a more accurate vision of public spending with the processes of organ donation.

The cost-benefit of having an exclusive professional in CIHDOTT is positive and the recovery of the invested funds is a short term aspect. The scarcity of economic studies in the area demonstrates the need for further research on these organ donation aspects. Further research could contribute to the efficient public policy implementation of this organ donation model, which has been strategically established in Ordinance of the National Transplant System since 2005, albeit poorly established in the Unified Health System (SUS).

## Footnotes

a Brasil. Lei nº 8.080, de 19 de setembro de 1990. Lei Orgânica da Saúde. Dispõe sobre as condições para a promoção, proteção e recuperação da saúde, a organização e o funcionamento dos serviços correspondentes e dá outras providências. *Diario Oficial Uniao*. 20 set 1990; Seção 1:018055.

b Ministério da Saúde. Portaria nº 531, de 30 de abril de 1999. *Diario Oficial Uniao.* 3 maio 1999; Seção 1: 8.

c Ministério da Saúde, Secretaria de Atenção à Saúde. Portaria nº 510, de 27 de novembro de 2010. Alterar, na Tabela de Procedimentos, Medicamentos e OPM do SUS, o valor dos procedimentos referentes às atividades de doação e transplante de órgãos.... Brasília (DF); 2010 [cited 2015 Apr 22]. Available from: http://bvsms.saude.gov.br/bvs/saudelegis/sas/2010/prt0510_27_11_2010.html

d DATASUS. Sistema de Gerenciamento da Tabela de Procedimentos, Medicamentos e OPM do SUS [cited 2014 Feb 8]. Available from: http://sigtap.datasus.gov.br/tabela-unificada/app/sec/inicio.jsp

e Conselho Federal de Enfermagem. Resolução COFEN-292/2004. Normatiza a atuação do enfermeiro na captação e transplante de órgãos e tecidos. Brasília (DF); 2004 [cited 2015 Apr 22]. Available from: http://www.cofen.gov.br/resoluo-cofen-2922004_4328.html

f Ministério da Saúde. Portaria n° 2.600, de 21 de outubro de 2009. Aprova o Regulamento Técnico do Sistema Nacional de Transplantes. Brasília (DF); 2009 [cited 2015 Apr 22]. Available from: http://bvsms.saude.gov.br/bvs/saudelegis/gm/2009/prt2600_21_10_2009.html

g Universidade de São Paulo, Faculdade de Arquitetura e Urbanismo. Análise de regressão: notas de aula. São Paulo; 2013 [cited 2013 Jan 28]. Available from: http://www.usp.br/fau/cursos/graduacao/arq_urbanismo/disciplinas/aut0516/Apostila_Regressao_Linear.pdf

h Ministério da Saúde. Portaria n° 2.600, de 21 de outubro de 2009. Aprova o Regulamento Técnico do Sistema Nacional de Transplantes. Brasília (DF); 2009 [cited 2015 Apr 22]. Available from: http://bvsms.saude.gov.br/bvs/saudelegis/gm/2009/prt2600_21_10_2009.html

i Brasil. Decreto-Lei nº 5.452, de 1º de maio de 1943. Aprova a Consolidação das Leis do Trabalho [cited 2015 Apr 14]^.^ Available from: http://www2.camara.leg.br/legin/fed/declei/1940-1949/decreto-lei-5452-1-maio-1943-415500-norma-pe.html

j Shinomata HO. Retorno sobre o investimento de um programa de atenção domiciliar em uma seguradora especializada em saúde [dissertation]. São Paulo (SP): Fundação Getúlio Vargas; 2007.

k SEBRAE. Prazo de retorno do Investimento (PRI). Brasília (DF); 2011 [cited 2014 Feb 9]. Available from: http://www.sebrae.com.br/sites/PortalSebrae/artigos/Prazo-de-Retorno-do-Investimento-%28PRI%29#0

l Portal Brasileiro de Dados Abertos. Taxa de câmbio - real (R$)/dólar americano 14/11/2013, Quinta-feira. 2015 [cited 2015 Aug 27]. Available from: http://dados.gov.br/dataset/taxa-de-cambio/resource/621ad4d8-c225-4785-bf75-0129b9b6423e

m Federação Nacional dos Médicos. Piso salarial médico. Brasília (DF); 2014 [cited 2014 Feb 3]. Available from: http://portal.fenam2.org.br/helper/show/400531#

n Sindicato dos Enfermeiros do Estado de São Paulo^. ^Negociações coletivas: pisos salariais para os enfermeiros do Estado de São Paulo de 1/9/2014 a 31/8/2015. São Paulo (SP); 2014 [cited 2014 Feb 3]. Available from: http://seesp.com.br/pisos/

o Vianna D. Economia da saúde. *Oxigênio Business Informativo* [Internet]. 2 ago 2010 [cited 2014 Feb 9]. Available from: http://www.oxigenioconsultores.com/pdfs/info_2010-08-02.pdf
