# Supplementary material for: Intra-hospital organ and tissue donation coordination project: cost-effectiveness and social benefits
Source: Rev Saude Publica. 2015 Oct 5;49:72. doi: 10.1590/S0034-8910.2015049005770 (PMC4603262; doi:10.1590/S0034-8910.2015049005770)
Supplement: Translation

# Translation: Projeto de coordenação intra-hospitalar de doação de órgãos: custo-efetividade e benefícios sociais

**Authors:** Vanessa Silva e Silva, Luciana Carvalho Moura, Renata Fabiana Leite, Priscilla Caroliny de Oliveira, Janine Schirmer, Bartira De’ Aguiar Roza

**Affiliations:** I Programa de Pós-Graduação em Enfermagem. Escola Paulista de Enfermagem. Universidade Federal de São Paulo. São Paulo, SP, Brasil; II Instituto Israelita de Responsabilidade Social. Hospital Israelita Albert Einstein. São Paulo, SP, Brasil; III Departamento de Enfermagem na Saúde da Mulher. Escola Paulista de Enfermagem. Universidade Federal de São Paulo. São Paulo, SP, Brasil; IV Departamento de Enfermagem Clínica e Cirúrgica. Escola Paulista de Enfermagem. Universidade Federal de São Paulo. São Paulo, SP, Brasil

**Keywords:** Coleta de Tecidos e Órgãos, Equipe de Assistência ao Paciente, Enfermagem, recursos humanos, Custos e Análise de Custo, Análise Custo-Benefício

## Abstract

**OBJETIVO:**

Avaliar a viabilidade de profissional especialista em comissões intra-hospitalares de doação de órgãos e tecidos para transplantes.

**MÉTODOS:**

Estudo epidemiológico, retrospectivo e transversal (2003 a 2011 e 2008 a 2012), realizado com dados de doação de órgãos para transplantes no estado de São Paulo. Foram avaliados nove hospitais (hospitais 1 a 9). Foram avaliadas, por regressão logística, diferenças em número de notificações de morte encefálica e doadores efetivos (variáveis dependentes) após a entrada do profissional especialista (variável independente) na comissão intra-hospitalar de doação de órgãos e tecidos para transplantes. Para avaliação do faturamento hospitalar, foram calculados o salário-hora de médico e enfermeiro, conforme legislação da Consolidação das Leis do Trabalho, o retorno e o prazo do retorno do investimento.

**RESULTADOS:**

Após a entrada de enfermeiro especialista na comissão, as notificações de morte encefálica e o número de doadores efetivos aumentaram no hospital 2 (4,17 e 1,52, respectivamente). No hospital 7, o número de doadores efetivos também aumentou de 0,005 para 1,54. E, após entrada de enfermeiro especialista, o faturamento hospitalar aumentou em 190,0% (variação de 40,0% a 1.955%). O custo mensal para 20 horas semanais do enfermeiro foi R$940,00 e, do médico, R$8.330,00. O retorno do investimento foi de 275%, em curto prazo (0,36 anos).

**CONCLUSÕES:**

A inserção de profissional especialista nas comissões intra-hospitalares para captação de doação de órgãos e tecidos para transplantes mostra-se custo-efetiva. Novos estudos econômicos na área podem contribuir com uma política pública eficiente de implantação desse modelo de captação de órgãos e tecidos para transplantes.

## INTRODUÇÃO

O Brasil possui o maior sistema público de saúde do mundo e, no caso dos transplantes, é único em relação ao modelo de financiamento. Isso se deve aos movimentos populares nas décadas de 1970/80 que estimularam a criação do Sistema Único de Saúde (SUS), em 1990, regulamentado pela Lei Orgânica da Saúde 8.080.^a^ O SUS foi definido como “conjunto de ações e serviços em todas as esferas (municipal, estadual e federal) da administração direta ou indireta, mantidas pelo Poder Público”.^14^

Após alguns anos de SUS, portarias e resoluções foram lançadas para instituir valores para o financiamento dos procedimentos de saúde. Entretanto, as ações referentes à doação e transplante de órgãos foram de fato beneficiadas em 1999, com a publicação da Portaria 531/GM,^b^ que criou o Fundo de Ações Estratégicas e Compensação (FAEC), complementadas, em 2010, com a Portaria 510,^c^ que alterou os valores referentes às atividades relacionadas ao transplante. Tais valores, armazenados em tabela unificada de procedimentos, medicamentos, órteses e próteses, são revisados e atualizados anualmente por meio de novas portarias.^d^

Com essa evolução histórica da legislação, houve aumento no orçamento financeiro público destinado às atividades relacionadas à doação e transplante de órgãos.

Apesar desses investimentos, alguns profissionais que atuam na área, e.g., em comissões intra-hospitalares de doação de órgãos e tecidos para transplantes (CIHDOTT), que não exercem essa atividade exclusivamente. São profissionais não remunerados para tal função e a exercem paralelamente a outras funções no hospital.

Na maioria dos casos, o coordenador da CIHDOTT é o enfermeiro, cuja atuação nessa área é regulamentada pela Resolução COFEN 292/2004.^e^ Essa resolução descreve como responsabilidade do enfermeiro a gestão de todo o processo de doação de órgãos, desde a identificação do potencial doador até a entrega do corpo à família.

Um hospital beneficente de São Paulo desenvolveu projeto de coordenação intra-hospitalar de doação de órgãos e tecidos para transplante em hospitais públicos, em parceria com o Sistema Nacional de Transplantes e a Secretaria de Saúde do Estado de São Paulo, em 2008, e realizado pelo Programa de Apoio ao Desenvolvimento Institucional do SUS.^12^

Esse projeto estimula o cumprimento da lei para a implantação de CIHDOTT no País,^f^ por meio da agregação de recursos humanos especializados. O objetivo é aumentar o número de doadores de órgãos e tecidos e, assim, diminuir os gastos com pacientes na fila de transplante. Para conhecer os resultados desta estratégia, foi realizada, após cinco anos, a avaliação do projeto.

O investimento real do projeto é a disponibilização de um profissional de saúde, especialista na área, sem custo para os hospitais selecionados que abrigam o projeto. Além disso, esses profissionais agregam mais valores à receita hospitalar, em decorrência do faturamento dos procedimentos relacionados à doação de órgãos, pagos pela tabela SUS.

O objetivo deste estudo foi avaliar a viabilidade de profissional especialista em comissões intra-hospitalares de doação de órgãos e tecidos para transplantes.

## MÉTODOS

Estudo epidemiológico, retrospectivo e transversal, realizado com dados de doação de órgãos da Secretaria de Saúde do Estado de São Paulo e do projeto de coordenação intra-hospitalar para doação de órgãos de hospital beneficente, de 2003 a 2012.

A população do estudo foi constituída pelas notificações de potenciais doadores e doadores efetivos do estado de São Paulo, com amostra antes e após chegada de enfermeiro especialista em nove hospitais, cujo critério de seleção foi ter, pelo menos, seis meses com coordenador intra-hospitalar contratado pelo projeto de coordenação intra-hospitalar para doação de órgãos do hospital beneficente.

A coleta de dados foi realizada em um único momento e os dados foram separados em dois grupos: um antes do início do projeto (setembro de 2003 a setembro de 2011) e outro após (maio de 2008 a dezembro 2012), com o mesmo número de meses avaliados antes e após a implantação do projeto em cada hospital.

O instrumento de coleta foi desenvolvido em planilha eletrônica (*software* Microsoft Excel^®^) com variáveis referentes ao número de notificações de morte encefálica e doadores efetivos nos nove hospitais que abrigaram o projeto, numerados de 1 a 9 para garantir o anonimato. Os números de notificações e doações efetivas foram transcritos do banco de dados do projeto de doação de órgãos para a planilha e foram validados com as informações oficiais do banco de dados da Secretaria de Saúde do Estado de São Paulo.

O instrumento foi construído tendo por base a experiência da pesquisadora e apresentado no grupo de estudos em doação de órgãos e tecidos para transplante da Universidade Federal de São Paulo (GEDOTT – Unifesp). Todas as sugestões foram consideradas para a elaboração do instrumento final.

Foi utilizada regressão logística, que estimou a influência do tempo (mês a mês) no número de notificações de morte encefálica e número de doadores efetivos. Tal análise permitiu analisar a influência da atuação do profissional nos resultados dos hospitais. O padrão de resposta diferenciado ocorreu após a chegada do enfermeiro especialista nos hospitais em questão, ou seja, este foi o marco zero da regressão.

Foi considerado modelo significante quando p < 0,05. Foi utilizado coeficiente de determinação (R^2^), medida de ajustamento de modelo estatístico linear generalizado em relação aos valores observados. O R^2^ varia entre 0 e 1 e indica a qualidade de ajuste do modelo: quanto mais próximo de 1, melhor o ajuste.^g^ Os resultados foram apresentados em gráficos lineares, que mostram a tendência de aumento (reta crescente) ou de diminuição (reta decrescente) dos eventos (notificações de morte encefálica e doadores efetivos).

Para a análise financeira, foram utilizadas informações do faturamento dos procedimentos de doação de órgãos pela Tabela SUS do Sistema de Gerenciamento da Tabela de Procedimentos, Medicamentos e OPM do SUS (SIGTAP^®^).^d^ Esses valores são de domínio público e foram multiplicados pelo número de doadores efetivos antes e depois do projeto, em todos os hospitais.

Foram considerados os seguintes procedimentos da tabela SUS, faturados pelas CIHDOTT, para o cálculo dos valores: avaliação de morte encefálica, coordenação de sala cirúrgica, diária de unidade de terapia intensiva (UTI), entrevista familiar e manutenção do potencial doador.

A portaria 2.600/2009 descreve em seu artigo 15 da seção II do capítulo III que:

[...] A CIHDOTT deverá [...] ser composta por, no mínimo, três membros integrantes de seu corpo funcional, dos quais um, que deverá ser médico ou enfermeiro, será o Coordenador Intra-Hospitalar de Doação de Órgãos e Tecidos para Transplante.

[...] § 2º Nos hospitais com CIHDOTT classificadas como II e III, conforme o art. 14 deste Regulamento, o Coordenador da Comissão Intra-Hospitalar de Doação de Órgãos e Tecidos para Transplante deverá possuir carga horária mínima de vinte horas semanais dedicadas exclusivamente à referida Comissão.

§ 3º O Coordenador da CIHDOTT classificada como III deverá ser obrigatoriamente um profissional médico. § [...].^h^

Sendo assim, as médias dos salários do médico e do enfermeiro em seus respectivos sindicatos foram pesquisadas. Os valores foram submetidos ao cálculo de salário-hora determinado pela Consolidação das Leis do Trabalho (CLT),^i^ para estimar o valor do pagamento para jornada de 20 horas semanais (80 horas/mês) desses profissionais, conforme segue:

Salário-hora = salário mês/nº horas de trabalho por dia × 30

Para o cálculo do número de horas de trabalho/dia, temos:

Horas de trabalho dia = horas de trabalho na semana/seis dias

(o salário mensal remunera seis dias e uma folga em uma semana)

O valor pago por mês para jornada de trabalho de 20 horas semanais é calculado multiplicando o valor salário-hora por 80 horas.

Em seguida, foi discutido se os valores da tabela SUS faturados poderiam custear um profissional com carga horária exclusiva nas comissões intra-hospitalares. Consideraram-se os salários com distribuição linear, sem cálculos específicos para análise de variações durante os meses ou anos. Isso por tratar-se de análise inicial do projeto, admitindo-se a limitação do estudo.

A utilização de métricas financeiras, como o retorno sobre investimento (*return on investment* – ROI) em estudos de programas de saúde, é discutida na literatura, refletindo análise básica de retorno dos investimentos em saúde. Não há intenção de ser análise aplicada, mas contribui para nortear os administradores de projetos, na área da saúde, quanto a sua continuidade.^j^ Serão necessárias análises mais aprofundadas para verificação de custos específicos, visto que a maioria dos estudos utiliza fórmulas adaptadas para a realidade estudada.^8^ [Bibr B9] ^-^ ^10^ ^,^ ^13^ ^,^ ^15^

O ROI pode ser definido pela fração: o numerador é o ganho, retorno, lucro ou benefício recebido como resultado do projeto, atividade ou sistema; enquanto o denominador é o custo (investimento) gasto para atingir o resultado.^3^

O investimento real para este projeto foi o recurso humano (convertido em Reais), estimado com base nos valores médios calculados para 20 horas semanais, segundo a CLT.

Os salários foram pagos aos profissionais com a verba destinada ao Programa de Apoio ao Desenvolvimento Institucional do SUS e não recaíram sobre a folha de pagamento dos hospitais selecionados. Podem ser entendidos como formas de investimentos, dependendo do contexto.^5^

O SUS não visa o lucro, mas o atendimento integral do usuário. Dependendo do valor do custo da realização dos procedimentos e dos valores de remuneração pagos, poderá haver lucro econômico ou não.^12^

O lucro líquido aqui considerado foi o resultado da subtração do faturamento dos procedimentos com a doação de órgãos e dos custos inerentes ao processo de doação; o primeiro representa o resultado direto do trabalho do profissional. Os custos tiveram por base o único estudo realizado na área que considerou todas as etapas do processo até a entrega do corpo para a família.^6^

O cálculo do ROI considera o lucro líquido dividido pelo custo do projeto. Quanto maior o ROI, maior o retorno do investimento, conforme a fórmula:^3^ ^,^ ^5^



A outra análise realizada foi o prazo para retorno do investimento (PRI):^k^



## RESULTADOS

Os modelos de regressão logística para as notificações de morte encefálica apresentaram baixo ajuste (p > 0,05), exceto para os hospitais 1, 2 e 5, por isso foram analisados descritivamente. Já para o número de doadores efetivos o modelo foi bem ajustado para os hospitais 2, 3 e 7, dos nove hospitais.

Houve significância estatística no aumento das notificações de morte encefálica e doadores efetivos a partir da entrada do enfermeiro especialista no hospital 2 (aumento de 4,17 notificações de morte encefálica e 1,52 doações efetivas comparado com o mês anterior), assim como para doadores efetivos no hospital 7 (aumento de 1,54).

Houve tendência crescente quanto às notificações de morte encefálica apenas para o hospital 9, sem significância estatística (Figuras 1 [Fig f02002] a 3). Os demais apresentaram tendência decrescente, com significância estatística nos hospitais 5 e 9.

Quanto ao número de doadores efetivos, tendência crescente foi observada em quatro hospitais (1, 2, 5 e 9) e, nos demais, tendência decrescente (3, 4, 6, 7 e 8), ambos sem significância estatística.

O faturamento hospitalar aumentou no período, quando considerada a soma de doadores efetivos mês a mês, multiplicada pelo valor correspondente da tabela SUS em reais e correspondente em dólares:^l^ R$259.593,97 de faturamento sem o projeto e R$745.704,44 após (diferença de R$486.110,47 ou 187,0%). O valor do lucro líquido foi de R$591.316,59. Houve aumento do faturamento hospitalar em todos os hospitais após a implantação do projeto, com variação de 40,0% a 1.955%.

Foi pesquisado o piso salarial médio médico e de enfermagem do estado de São Paulo, conforme descrito nos métodos. O piso salarial do médico com jornada de 20 horas semanais, segundo a Federação Nacional dos Médicos (FENAM),^m^ era de R$10.412,00 por mês. O do enfermeiro, calculado pela média dos pisos salariais dos sindicatos do estado de São Paulo,^n^ era R$2.115,61 para 36 horas semanais.

O salário-hora, segundo as normas da CLT, foi de:

Salário-hora médico = R$10.412,00/100 h = R$104,12/h

Salário-hora enfermeiro = R$2.115,61/180 h = R$11,75/h

O coordenador de uma CIHDOTT tipo II pode ser um enfermeiro, logo o custo mensal seria de R$940,00 para 20 horas semanais.

Para CIHDOTT tipo III, teríamos custo mensal de R$8.329,60 para manter um coordenador médico atuando por 20 horas semanais exclusivas.

Nos hospitais estudados, os profissionais eram enfermeiros. Portanto, o valor total do investimento salarial foi de R$215.260,00, considerando o número de meses do projeto em cada hospital (56 meses no hospital 1; 39 no hospital 2; 8 no hospital 3; 17 no hospital 4; 20 no hospital 5; 20 no hospital 6; 26 no hospital 7; 28 no hospital 8; e 15 no hospital 9).

Com os valores de investimento e do lucro líquido apresentados, o ROI e o PRI foram:



## DISCUSSÃO

Os resultados de efetivação de doadores nos hospitais após a entrada do profissional foram positivos. Isso confirma os achados de publicações que discutem o bom funcionamento de uma CIHDOTT como de suma importância para a melhoria nos números de doadores efetivos.^16^

A melhoria nos processos envolvidos com a doação de órgãos por meio da presença da CIHDOTT garante melhor qualidade e quantidade de órgãos fornecidos ao sistema público de transplantes do País.^2^

A melhoria dos números de notificação de morte encefálica e de doadores efetivos pode garantir a equidade, princípio norteador do SUS, e o retorno do paciente ao mercado de trabalho, visto que muitos dependem de benefícios estatais para subsistir durante a fase pré-transplante. Adicionalmente, como os custos indiretos da não realização de transplantes são elevados,^4^ ^,^ ^11^ essa melhoria ainda diminuiria os gastos públicos em saúde, possibilitando realocação de recursos nos hospitais para serem aplicados em setores como as comissões intra-hospitalares de doação de órgãos e tecidos para transplantes.

Apesar de o padrão de número de notificações de morte encefálica ou de doadores efetivos ter se mantido mais elevado do que no período anterior à implantação do projeto, houve tendência decrescente da reta, com exceção de quatro hospitais.

A permanência prolongada no mesmo hospital, como observado nos hospitais 1 e 2, pode proporcionar desgaste do profissional. Estudos relacionam altas taxas de síndrome de Burnout com os profissionais da área de captação de órgãos, podendo variar de exaustão emocional, despersonalização à reduzida realização pessoal.^1^

Apoio psicológico e rodízio dos profissionais entre os diferentes hospitais são necessários para que a realidade da morte não seja o principal aspecto analisado, e sim a prevalência da vida e dos aspectos positivos que a atividade de captação de órgãos proporciona.^7^

A presença do coordenador com carga horária e remuneração exclusivas para as atividades de doação de órgãos é realidade em poucos locais do País, como no estado de São Paulo, com o projeto apresentado neste estudo. No entanto, apesar dessas iniciativas, não há regulamentações que sustentem a remuneração, pelos hospitais ou pelo governo, dos coordenadores intra-hospitalares.

Adicionalmente, o diagnóstico de morte encefálica representa morte do indivíduo. Os custos são altos para manter um doador de órgãos, com equipe especialista, e insumos hospitalares. Para isso, o SUS criou o pagamento da diária de UTI para potenciais doadores de órgãos, visando garantir o custeio desta etapa *post mortem* do potencial doador.

O profissional em uma CIHDOTT acompanha os casos de morte encefálica desde a busca ativa dos pacientes em coma, nível 3 na escala de coma de Glasgow, até a efetivação ou não do potencial doador. Isso facilita o acolhimento familiar, garante o direito de decisão da família em relação à doação de órgãos e tecidos (de acordo com a vontade do falecido) e melhora a rotatividade de leitos hospitalares.

A rotatividade de leitos nesses casos refere-se a dois desfechos: na doação positiva, a liberação do leito ocorre quando o doador deixa a UTI para ser submetido à cirurgia de extração multiorgânica; já na negativa familiar, o leito é liberado após serem suspensos os suportes terapêuticos pelo médico de plantão para entrega do corpo à família, para seguirem com as cerimônias fúnebres. Ambos os casos apresentam benefício social, visto que há disponibilização de um leito, o que garante a possibilidade terapêutica a outro indivíduo. Além disso, quanto menor o tempo para o desfecho, menor o custo com recursos humanos, medicações, aparelhos, gases terapêuticos, entre outros.

O faturamento hospitalar é outro ponto a ser ressaltado, pois o SUS destina verba específica para pagamento dos procedimentos relacionados à doação de órgãos. Assim, o profissional treinado poderá auxiliar na realização do faturamento hospitalar e aumentar os valores arrecadados para o hospital.

O faturamento dos procedimentos de doação de órgãos nos hospitais estudados contribuiu para a receita hospitalar em pelo menos 40,0% a mais que antes da entrada do profissional na CIHDOTT. Tal benefício poderá ser mais bem explicitado em futuras análises que utilizem ferramentas da Economia da Saúde como a análise de custo-efetividade, visto que “uma intervenção em saúde é dita custo-efetiva se produz um benefício clínico justificável para seu custo”.^o^

A doação de órgãos deve ser autossustentável para compensar o benefício social para o indivíduo que tem apenas essa possibilidade terapêutica. O faturamento hospitalar advindo dos procedimentos realizados com os potenciais doadores e doadores efetivos de órgãos (diária de UTI, entrevista familiar, coordenação de sala cirúrgica, entre outros) poderia ter como objetivo custear os próprios gastos com o potencial doador (mão de obra dos profissionais de enfermagem, uso de medicamentos, uso de gases terapêuticos, entre outros) e com a contratação de um profissional exclusivo para exercer a coordenação intra-hospitalar.

Com a cobrança de todos os itens listados no método, o doador poderia gerar faturamento de R$2.228,63, segundo tabela SUS. Assim, considerando apenas os gastos com o vencimento salarial de um médico coordenador para os casos de CIHDOTT tipo III, seria necessária média de 3,7 doadores por mês com a cobrança de todos os itens listados anteriormente. Um doador por mês seria o suficiente para custear seus vencimentos para 20 horas semanais para o custeio de um enfermeiro coordenador de CIHDOTT tipo II.

A contratação de enfermeiros para coordenar CIHDOTT tipo III poderia ser uma alternativa economicamente viável para esses hospitais, com base na Economia da Saúde. A estrutura do sistema de transplantes do Estado de São Paulo conta com apoio dos Serviços de Procura de Órgãos, que têm como coordenadores profissionais médicos que validam os doadores de órgãos e auxiliam durante o processo de doação. Além disso, muitas atividades desempenhadas pelos profissionais da CIHDOTT são de competência do enfermeiro, o que garantiria a segurança e qualidade do processo no âmbito hospitalar.

Os gastos com o vencimento mensal desse coordenador para jornada de 20 horas semanais representariam 11,3% do salário médico. Para tanto, seriam necessários estudos apurados e possíveis mudanças na legislação vigente.

Nos hospitais com coordenadores enfermeiros, houve eficiência na utilização dos fundos aplicados nos vencimentos salariais dos enfermeiros do projeto, com ROI de 275,0% e baixo prazo para retorno dos investimentos: menos de um ano (PRI = 0,36 anos). Tais indicadores econômicos avaliam o impacto financeiro e são úteis para a tomada de decisão dos gestores públicos. Entretanto, precisam ser aplicados de maneira mais completa em novos estudos para se ter visão mais fidedigna dos gastos públicos com os processos de doação de órgãos.

O custo-benefício de ter profissional exclusivo na CIHDOTT é positivo e a recuperação dos valores investidos ocorre em curto prazo. A escassez de estudos econômicos na área mostra a necessidade de novas investigações acerca de tais aspectos da doação de órgãos. Isso poderia contribuir com política pública eficiente de implantação deste modelo de captação de órgãos, estrategicamente estabelecido em Portaria do Sistema Nacional dos Transplantes desde 2005, mas precariamente instituído no Sistema Único de Saúde.

**Figura 1:** Número de notificações de morte encefálica antes e depois do projeto no Hospital 2. São Paulo, 2005 a 2011.

**Figura 2 f02002:** Número de doadores efetivos antes e depois do projeto no Hospital 2. São Paulo, 2005 a 2011.

**Figura 3:** Número de doadores efetivos antes e depois do projeto no Hospital 1. São Paulo, 2003 a 2012.

## Footnotes

a Brasil. Lei nº 8.080, de 19 de setembro de 1990. Lei Orgânica da Saúde. Dispõe sobre as condições para a promoção, proteção e recuperação da saúde, a organização e o funcionamento dos serviços correspondentes e dá outras providências. *Diario Oficial Uniao*. 20 set 1990; Seção 1:018055.

b Ministério da Saúde. Portaria nº 531, de 30 de abril de 1999. *Diario Oficial Uniao.*3 maio 1999; Seção 1: 8.

c Ministério da Saúde, Secretaria de Atenção à Saúde. Portaria nº 510, de 27 de novembro de 2010. Alterar, na Tabela de Procedimentos, Medicamentos e OPM do SUS, o valor dos procedimentos referentes às atividades de doação e transplante de órgãos.... Brasília (DF); 2010 [citado 2015 abr 22]. Disponível em: http://bvsms.saude.gov.br/bvs/saudelegis/sas/2010/prt0510_27_11_2010.html

d DATASUS. Sistema de Gerenciamento da Tabela de Procedimentos, Medicamentos e OPM do SUS [citado 2014 fev 8]. Disponível em: http://sigtap.datasus.gov.br/tabela-unificada/app/sec/inicio.jsp

e Conselho Federal de Enfermagem. Resolução COFEN-292/2004. Normatiza a atuação do enfermeiro na captação e transplante de órgãos e tecidos. Brasília (DF); 2004 [citado 2015 abr 22]. Disponível em: http://www.cofen.gov.br/resoluo-cofen-2922004_4328.html

f Ministério da Saúde. Portaria n° 2.600, de 21 de outubro de 2009. Aprova o Regulamento Técnico do Sistema Nacional de Transplantes. Brasília (DF); 2009 [citado 2015 abr 22]. Disponível em: http://bvsms.saude.gov.br/bvs/saudelegis/gm/2009/prt2600_21_10_2009.html

g Universidade de São Paulo, Faculdade de Arquitetura e Urbanismo. Análise de regressão: notas de aula. São Paulo; 2013 [citado 2013 jan 28]. Disponível em: http://www.usp.br/fau/cursos/graduacao/arq_urbanismo/disciplinas/aut0516/Apostila_Regressao_Linear.pdf

h Ministério da Saúde. Portaria n° 2.600, de 21 de outubro de 2009. Aprova o Regulamento Técnico do Sistema Nacional de Transplantes. Brasília (DF); 2009 [citado 2015 abr 22]. Disponível em: http://bvsms.saude.gov.br/bvs/saudelegis/gm/2009/prt2600_21_10_2009.html

i Brasil. Decreto-Lei nº 5.452, de 1º de maio de 1943. Aprova a Consolidação das Leis do Trabalho [citado 2015 abr 14]. Disponível em: http://www2.camara.leg.br/legin/fed/declei/1940-1949/decreto-lei-5452-1-maio-1943-415500-norma-pe.html

j Shinomata HO. Retorno sobre o investimento de um programa de atenção domiciliar em uma seguradora especializada em saúde [dissertação]. São Paulo (SP): Fundação Getúlio Vargas; 2007.

k SEBRAE. Prazo de retorno do Investimento (PRI). Brasília (DF); 2011 [citado 2014 fev 9]. Disponível em: http://www.sebrae.com.br/sites/PortalSebrae/artigos/Prazo-de-Retorno-do-Investimento-%28PRI%29#0

l Portal Brasileiro de Dados Abertos. Taxa de câmbio - real (R$)/dólar americano 14/11/2013, Quinta-feira. 2015 [citado 2015 ago 27]. Disponível em: http://dados.gov.br/dataset/taxa-de-cambio/resource/621ad4d8-c225-4785-bf75-0129b9b6423e

m Federação Nacional dos Médicos. Piso salarial médico. Brasília (DF); 2014 [citado 2014 fev 3]. Disponível em: http://portal.fenam2.org.br/helper/show/400531#

n Sindicato dos Enfermeiros do Estado de São Paulo^. ^Negociações coletivas: pisos salariais para os enfermeiros do Estado de São Paulo de 1/9/2014 a 31/8/2015. São Paulo (SP); 2014 [citado 2014 fev 3]. Disponível em: http://seesp.com.br/pisos/

o Vianna D. Economia da saúde. *Oxigênio Business Informativo* [Internet]. 2 ago 2010 [citado 2014 fev 9]. Disponível em: http://www.oxigenioconsultores.com/pdfs/info_2010-08-02.pdf
